# OA05.01. Altering nutrition-related behaviors of healthcare professionals through CME involving nutrition experts and chefs

**DOI:** 10.1186/1472-6882-12-S1-O17

**Published:** 2012-06-12

**Authors:** D Eisenberg, A Myrdal Miller, K McManus, M Erickson, G Drescher, J Burgess, A Bernstein, B Rosner, E Rimm, W Willett

**Affiliations:** 1 Harvard Medical School, Chestnut Hill, USA; 2 Culinary Institute of America, St. Helena, USA; 3 Brigham and Women's Hospital, Boston, USA; 4 Cleveland Clinic, Cleveland, USA; 5 Harvard School of Public Health, Boston, USA

## Purpose

For healthcare professionals, practicing a healthful behavior oneself is a powerful predictor of counseling patients about these same self-care behaviors (e.g. exercise, wearing seat belts, etc). We explored the feasibility of altering healthcare professionals’ personal and professional behaviors with regard to diet, food preparation, and their ability to advise patients who are overweight or obese as a result of a four-day continuing medical education (CME) conference, “Healthy Kitchens, Healthy Lives–Caring for Our Patients and Ourselves,” which combines both medical and culinary education.

## Methods

This CME program occurs in a facility that allows for didactic presentations, cooking demonstrations, and hands-on cooking workshops. An anonymous survey of conference registrants’ (n=387) nutrition-related behaviors was conducted at the start of the conference, March 2010, and twelve weeks later. The CME program included plenary lectures, culinary demonstrations, hands-on cooking sessions, interactive workshops, case presentations, meals, and tastings. Main outcome measures were self-reported changes in nutrition-related personal and professional behaviors.

## Results

Of 387 registrants, 219 (57%) completed the survey at baseline and 192 (50%) completed the follow up survey at twelve weeks. 265 (66%) registrants were physicians. Respondents reported significant positive changes in (1) frequency of cooking their own meals (p<0.001), (2) personal awareness of calorie consumption (p≤0.05), (3) frequency of vegetable, nut, and whole grain consumption (p≤0.04), (4) ability to formally address a patient’s nutrition status (p<0.001), and (5) ability to successfully advise overweight/obese patients regarding specific nutritional/lifestyle habits (p<0.001).

## Conclusion

It is possible to alter healthcare professionals’ personal and professional nutrition-related behaviors using a four-day continuing educational program involving a combination of medical and culinary education in an interactive setting. In light of increasing rates of obesity and diabetes, how this multi-disciplinary educational approach can alter the behaviors of large numbers of healthcare practitioners and, hypothetically, their patients, should be explored further.

